# Biocompatibility of Platinum Nanoparticles in Brain *ex vivo* Models in Physiological and Pathological Conditions

**DOI:** 10.3389/fnins.2021.787518

**Published:** 2021-12-15

**Authors:** Maurizio Gulino, Sofia Duque Santos, Ana Paula Pêgo

**Affiliations:** ^1^i3S – Instituto de Investigação e Inovação em Saúde, Universidade do Porto, Porto, Portugal; ^2^INEB – Instituto de Engenharia Biomédica, Universidade do Porto, Porto, Portugal; ^3^FEUP – Faculdade de Engenharia da Universidade do Porto, Porto, Portugal; ^4^ICBAS – Instituto de Ciências Biomédicas Abel Salazar, Universidade do Porto, Porto, Portugal

**Keywords:** metallic nanoparticles, cytotoxicity, inflammation, gliosis, neurodegeneration, brain tissue explants

## Abstract

Platinum nanoparticles (PtNPs) have unique physico-chemical properties that led to their use in many branches of medicine. Recently, PtNPs gathered growing interest as delivery vectors for drugs, biosensors and as surface coating on chronically implanted biomedical devices for improving electrochemical properties. However, there are contradictory statements about their biocompatibility and impact on target organs such as the brain tissue, where these NPs are finding many applications. Furthermore, many of the reported studies are conducted in homeostasis conditions and, consequently, neglect the impact of the pathologic conditions on the tissue response. To expand our knowledge on the effects of PtNPs on neuronal and glial cells, we investigated the acute effects of monodisperse sodium citrate-coated PtNPs on rat organotypic hippocampal cultures in physiological or neuronal excitotoxic conditions induced by kainic acid (KA). The cellular responses of the PtNPs were evaluated through cytotoxic assays and confocal microscopy analysis. To mimic a pathologic scenario, 7-day organotypic hippocampal cultures were exposed to KA for 24 h. Subsequently, PtNPs were added to each slice. We show that incubation of the slices with PtNPs for 24 h, does not severely impact cell viability in normal conditions, with no significant differences when comparing the dentate gyrus (DG), as well as CA3 and CA1 pyramidal cell layers. Such effects are not exacerbated in KA-treated slices, where the presence of PtNPs does not cause additional neuronal propidium iodide (PI) uptake in CA3 and CA1 pyramidal cell layers. However, PtNPs cause microglial cell activation and morphological alterations in CA3 and DG regions indicating the establishment of an inflammatory reaction. Morphological analysis revealed that microglia acquire activated ameboid morphology with loss of ramifications, as a result of their response to PtNPs contact. Surprisingly, this effect is not increased in pathological conditions. Taken together, these results show that PtNPs cause microglia alterations in short-term studies. Additionally, there is no worsening of the tissue response in a neuropathological induced scenario. This work highlights the need of further research to allow for the safe use of PtNPs. Also, it supports the demand of the development of novel and more biocompatible NPs to be applied in the brain.

## Introduction

For many decades, platinum has been employed in many branches of nanobiomedicine (Jeyaraj et al., 2019).

This metal presents prodigious physico-chemical characteristics such as good electrical conductivity, chemical stability and surface functionalization, that makes it unique among all the noble metals and suitable for several biomedical applications (Fahmy et al., 2020).

Platinum nanoparticles (PtNPs) have recently gained growing interest over Pt derived compounds for the treatment of several types of cancers, thanks to their ability to induce apoptosis and cell cycle arrest, genotoxicity, oxidative stress and morphological changes in the structure of the tumor tissue. In accordance, PtNPs have been shown to inhibit proliferation of human lung cancer cells (Bendale et al., 2016), breast cancer cells (Şahin et al., 2018) glioblastoma and neuroblastoma cancer cells (Asharani et al., 2010; Kutwin et al., 2017a,b; Gurunathan et al., 2020), among others.

PtNPs have been also proposed for photothermal treatment of certain types of tumors, as they can adsorb light and convert it into heat energy causing apoptosis of cancer cells and tumor regression (Manikandan et al., 2013; Samadi et al., 2018; Chang et al., 2019; Depciuch et al., 2020). By means of their surface functionalization, PtNPs have been further explored as promising materials for drug delivery applications (Shim et al., 2017; Cao et al., 2018; Mukherjee et al., 2020). Moreover, substantial research has been conducted on the development of surface functionalized PtNPs to confer enzymatic activity as well as for improving biocompatibility, bioavailability and stability in the context of their medical use (He et al., 2014; Deng et al., 2017; Franco-Ulloa et al., 2020). Remarkable attention has also been gathered by the employment of PtNPs in microelectrode design thanks to their adaptive electrochemical and mechanical properties that make them suitable for a variety of new design approaches of microelectrodes with superior properties (Islam and Islam, 2013; Aggas et al., 2019; Moeini et al., 2021). Overall, PtNPs have proven to be innovative tools to meet the requirements of sensitivity, target specificity and miniaturization of new designed microelectrodes. Despite the promising results obtained from early preclinical studies, there is still uncertainty in the scientific community regarding Pt toxicity and accumulation in organs and tissues (Kovach et al., 2016; Labrador-Rached et al., 2018). Conflicting findings are present in the literature about their impact in terms of safety (Wissel et al., 2018; Czubacka and Czerczak, 2019; Zeng et al., 2020; Shepherd et al., 2021). *In vivo* studies conducted on rats reported inflammation, cellular alterations in organs and negative effects on post-natal development (Adeyemi et al., 2016; Park et al., 2016). On the other hand, no adverse effects such as increased reactive gliosis or altered neuronal counts were observed 3 weeks after neural implants of nano-coated electrodes with PtNPs (Angelov et al., 2016). However, few studies have focused on the PtNPs impact on the brain tissue in a pathological scenario (Prasek et al., 2013; Adeyemi et al., 2018). Considering the increasing number of applications of PtNPs for implantable microelectrode design, it is paramount to investigate their impact on neuronal and glial cells, and to conduct biocompatibility tests not only in homeostatic conditions, but also under induced pathological conditions to mimic the clinical scenario, where these materials are mainly employed. A compromised brain tissue unavoidably displays structural and cellular alterations that could lead to a different response compared to a brain tissue under physiological conditions. In this study, we selected organotypic hippocampal cultures as a model to investigate the biocompatibility of PtNPs and assess the acute response of the brain tissue in terms of cytotoxicity and cellular response. These cultures were incubated with monodisperse citrate-coated PtNPs under normal and pathological excitotoxic conditions induced by kainic acid (KA) treatment (Routbort et al., 1999). These stabilized PtNPs were assessed for the cellular responses occurring after short-time contact with brain tissue through cytotoxic and microscopy analysis, focusing on the morphological alterations of neurons, microglia, and astrocytes.

## Materials and Methods

### Animals

All the experiments were performed according to National and European Union guidelines (EU Directive 2010/63/EU for animal experiments) and with the utmost care to minimize animal suffering as well as the number of animals used. All the procedures using animals were approved by the i3S Ethics Committee (CEA, i3S). Animals were kept in an enriched housing environment with *ad libitum* feed and water supply. They were kept under a 12 h light/12 h dark cycle in the i3S animal facility with controlled ambient temperature and humidity. Wistar Han rat pups, males and females, 6–8 days old were used to prepare the hippocampal slice cultures.

### Organotypic Hippocampal Slice Cultures

Organotypic hippocampal slice cultures were prepared as previously described (Stoppini et al., 1991). Briefly, rat pups were sacrificed by decapitation. The brain was extracted and immediately immersed in sterile ice-cold low Na^+^ artificial cerebrospinal fluid (aCSF) solution containing 1 mM CaCl_2_, 10 mM D-glucose, 4 mM KCl, 5 mM MgCl_2_, 26 mM NaHCO_3_, and 234 mM sucrose, previously bubbled for 15–20 min with 95% O_2_ and 5% CO_2_, as described by Opitz-Araya and Barria (2011). The brain was cut longitudinally, and both hippocampi extracted, and transversally cut using a McIlwain Tissue chopper (EC 350, Campden Instruments). 400 μm slices were obtained and gently separated using small spatulas under a Stereomicroscope (Leica S8APO). Only intact slices were selected and transferred into a 0.4 μm Millicell^®^ PTFE culture inserts (Merck-Millipore), placed in multi-6 well plates (Corning). Each well contained 1 ml of incubation medium composed of Opti-MEM™ (Gibco) based medium supplemented with 25% (v/v) heat inactivated horse serum (Gibco), 25% (w/v) Hank’s balanced salt solution (Gibco), glucose 25 mM (Sigma Aldrich), and penicillin/streptomycin (Gibco, 100 μg/ml). Slices were maintained at 37°C with 5% CO_2_ and 95% atmospheric air for 7 days. Medium was changed 24 h after the plating and then after every 2 days. From each animal, 25–30 hippocampal slices were selected and used for one set of experiments.

### Kainic Acid-Induced Excitotoxicity and Platinum Nanoparticles Administration

**FIGURE 1:**
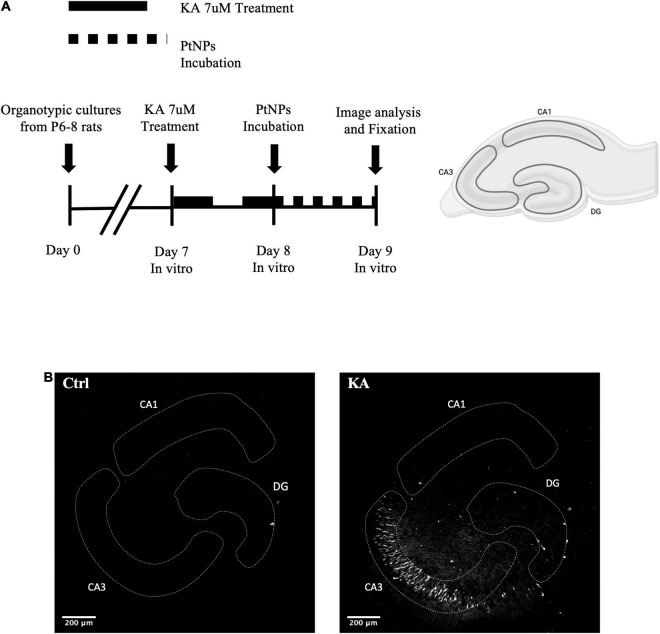
Establishment of rat hippocampal organotypic cultures as a 3D model to study the biocompatibility of Platinum nanoparticles (PtNPs) both in normal and pathological conditions. **(A)** Experimental scheme for inducing the pathological state and assessing the biocompatibility of PtNPs in the organotypic hippocampal slices. After 7 days in culture, slices are treated with kainic acid (KA) for 24 h followed by a 24-h incubation period with PtNPs. **(B)** Representative Fluoro-jade C staining of degenerating neurons in organotypic hippocampal slices under control condition and KA 7 uM at day 8 *in vitro*.

At day 7 of culture, excitotoxicity was induced by incubation of the slices with KA at a concentration of 7 μM (Abcam) diluted in 1 ml of fresh incubation medium for 24 h, in an adaptation of an experimental protocol from Järvelä et al. (2011). Subsequently, monodisperse citrate coated PtNPs (Nanocomposix, 50 nm) were added, as received, on the top of each slice and incubated for 24 h. The experimental plan is shown in Figure 1A. PtNPs administration was carried out using a Narishige Pneumatic Microinjector (Model IM-300) connected to a nitrogen cylinder. Each insert was transferred to a petri dish, containing 2 ml of warm incubation medium. 200 ng of PtNPs suspended in aqueous 2 mM sodium citrate buffer, at a concentration of 1 mg/ml were loaded into glass microcapillaries (WPI 1B200F-4, with Filament) inserted in a three-dimensional manipulator. 12–15 drops were deposited on the CA3, CA1, and dentate gyrus (DG) areas, without touching the tissue. In control slice cultures, sodium citrate buffer was deposited in the same way. All the procedures were carried out in sterile conditions inside a laminar flow hood equipped with a stereomicroscope. After the procedure, the medium was replaced with fresh pre-heated medium.

### Propidium Iodide Uptake

Cell viability was assessed by propidium iodide (PI) exclusion, to evaluate the impact of PtNPs on the slices under normal and excitotoxic conditions. 24 h after NPs administration, PI (Gibco) was added to the culture medium for 1 h at 4 μg/ml. Slices treated with Triton X-100 0.1% (v/v) (Sigma Aldrich) in phosphate buffered saline (PBS) for 1 h prior to PI incubation were used as positive controls. For visualization, each membrane insert was gently cut out and placed inside a 35 mm imaging μ-dish (ibidi) as previously described (Seidl and Rubel, 2010).

### Fluoro-Jade Staining

Fluoro-jade C staining was used to assess the neuronal degeneration caused by excitotoxicity induced with KA. After KA incubation for 24 h, the membrane inserts were fixed in 4% (w/v) paraformaldehyde (PFA, Sigma Aldrich) for 1 h with gentle shaking at room temperature (RT). After fixation, the membranes with slices were incubated in 0.06% (w/v) KMnO_4_ for 5 min (Sigma Aldrich) prior to staining with 0.001% (w/v) Fluoro-Jade C solution for 30 min (Merck Millipore). Subsequently, the membranes were transferred onto glass slides and dried at RT for 5–10 min. Finally, slices were cleared in xylene (Sigma Aldrich) and coverslipped with DPX mounting medium (Merck).

### Immunofluorescence

At the end of the incubation period with PtNPs, slices were fixed in 4% (w/v) PFA for 1 h with gentle shaking at RT. Slices were incubated in permeabilization/blocking buffer composed of 1% (w/v) bovine serum albumin (BSA, Sigma Aldrich), 5% (v/v) heat-inactivated fetal bovine serum (FBS, Gibco) and 1% (v/v) Triton X-100 in PBS overnight at 4°C with gentle shaking. After PBS washing, slices were incubated with rabbit anti-ionized calcium binding adaptor molecule 1 (Iba-1, 1:800, Wako Chemicals), mouse anti-β3-tubulin (1:500, Promega) and rabbit anti-glial fibrillary acidic protein (GFAP, 1:500, Abcam) for 48 h at 4°C with gentle shaking diluted in blocking buffer containing 1% (w/v) BSA, 5% (v/v) FBS, and 0.3% (v/v) Triton X-100 in PBS for 48 h at 4°C with gentle shaking. Slices were washed again with 0.05% (v/v) Triton X-100 in PBS at RT with gentle shaking. After primary antibody incubation, slices were incubated with Alexa Fluor 488 rabbit anti-goat IgG (H + L) secondary antibody (Invitrogen) and Alexa Fluor 647 donkey anti-mouse IgG (H + L) secondary antibody (Invitrogen) for 3 h at RT with gentle shaking and then washed again with 0.05% Triton-100 in PBS at RT with gentle shaking.

To stain the nuclei, slices were incubated in Hoechst 33342 solution (1 μg/ml, Thermo Fisher Scientific) in PBS for 30 min. Slices were placed on glass slides for mounting, as previously described (Yoon et al., 2011), using a bridge mounting technique to preserve integrity of the slices. Briefly, two 22 mm × 22 mm coverslips were glued on the glass slides to create a chamber in which to place the slices. The space created was filled with Mowiol/glycerol (3:1, Sigma Aldrich) mounting medium and gently covered with a 40 mm × 22 mm coverslip to close the gap between the two slides. The coverslip was glued with nail varnish and stored in the dark at 4°C, till further use.

### Image Analysis and Quantification

For image acquisition of PI uptake, a Zeiss Axiovert 200M inverted fluorescence microscope equipped with a 565 nm filter was used. Images were acquired using a 5X objective. Analysis and quantification of PI uptake was performed in CA3, CA1, and DG regions of each brain slice using Image J software (version 1.53; National Institutes of Health, United States).

Fluoro-jade C and immunofluorescence image acquisition was performed using a Leica Scanning Confocal SP8 equipped with 405, 488, and 633 filters (Leica Microsystems, Germany) composed of an inverted microscope Leica DMI8 (Leica Microsystems, Germany) and LAS X software (Leica Microsystems, Germany). For fluorescence intensity quantification, images were acquired using 10X objective. Confocal image stacks of 512 × 512 (16-bit depth, pixel size 3 μm, and zoom 0.75). Quantification of Iba-1, GFAP, β3-tubulin and PI intensity in the CA3, CA1, and DG regions was performed using Image J software. 80–100 μm stacks were acquired from each slice starting from the top of the tissue.

For the image acquisition of Iba-1, GFAP and β3-tubulin densities in CA3 region, images were acquired using a 40X objective, with confocal image stacks of 1,024 × 1,024 pixel, 16-bit depth, pixel size 0.63 μm, and zoom 1.0. Quantification was performed using the surface analysis tool on Imaris Software (version 9.5; Oxford Instruments, United Kingdom).

For the of 3D- reconstruction of microglial cell morphology, images were acquired with higher detail using 63X objective, with confocal 16-bit depth image stacks of 1,024 × 1,024 pixels, with pixel size of 0.334 μm and zoom set at 3.0. Single cells with complete and defined processes were selected and imaged for analysis. Quantification of morphology was performed using the filament tool tracer on Imaris software. Cell processes were automatically created from the cell soma using AutoPath (no loops) algorithm and manually adjusted for a proper tracing. The number of processes as well as the number of terminal points and branching points was calculated per each cell. To quantify the degree of arborization, the total number of Sholl intersections and the filament full branch depth (the maximum number of branches between a starting point and a terminal point) was also calculated (Salois and Smith, 2016).

### Statistics

Data from three independent experiments were presented as the mean ± standard error of the mean (SEM). Tukey’s test after one-way analysis of variance (ANOVA) was used for statistical analysis. Analysis and visualization of data was carried out using GraphPad Prism 9 (GraphPad Software, San Diego, CA, United States).

## Results

### Organotypic Hippocampal Slices Are Responsive to the Pathological Stimulus

**FIGURE 2:**
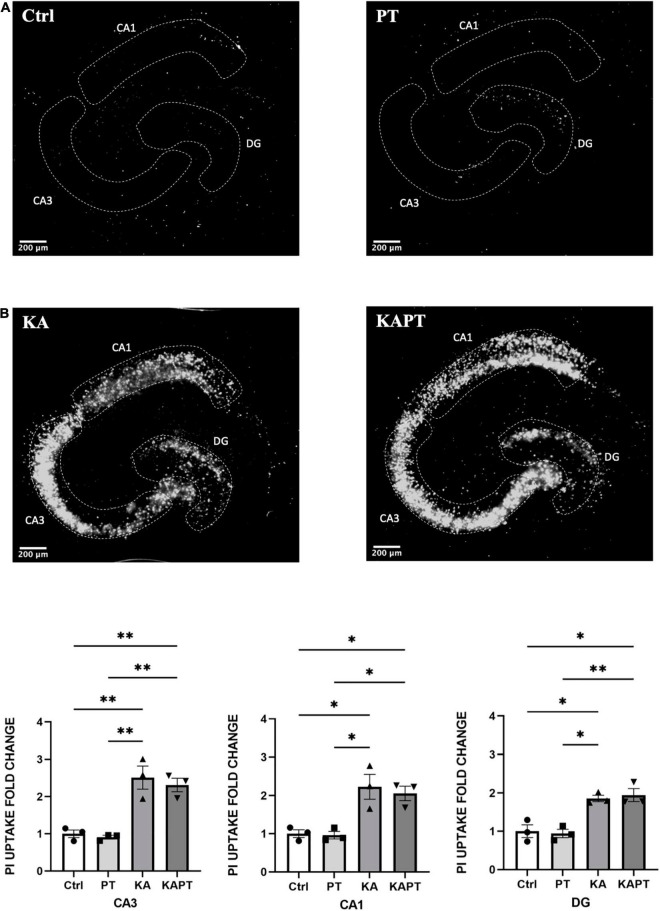
Platinum nanoparticles (PtNPs) do not impact cell viability in normal and pathological conditions after 24 h of contact. **(A)** Representative propidium iodide (PI) staining of dead cells in organotypic hippocampal slices under control conditions (CTRL; untreated slices), PtNPs (PT), kainic acid treatment (KA), and KA treatment + PtNPs (KAPT). **(B)** Quantitative analysis of PI uptake in CA3, CA1, and dentate gyrus (DG) in normal and pathological conditions. Data are expressed as integrated density fold change from the basal value of control slices and presented as mean ± standard error of the mean (SEM) (*n* = 3 independent experiments; 4–6 slices analyzed per group/experiment). Tukey’s test after one–way analysis of variance (ANOVA) was used for statistical analysis. *p < 0.05; ^**^p < 0.01.

To determine the response of the organotypic hippocampal slice cultures to excitotoxic damage induced by KA, we first quantified the uptake of PI to assess cell viability after the treatment. As previously reported (Holopainen et al., 2004; Järvelä et al., 2011; Jung et al., 2013; Kim et al., 2015), treatment with KA at low concentrations (7 μM) for 24 h caused significant neural cell viability impairment in CA3 and, to a lower extent, in CA1 and DG regions compared to untreated cultures (Figure 2A). To confirm the cell population that was most impacted, we performed Fluoro-jade C staining to assess neuronal degeneration. Imaging of organotypic hippocampal slices showed staining of degenerating neurons in the CA3 region, with the same staining pattern described in previous works (Figure 1B; Jung et al., 2013). These results confirmed the expected response of the organotypic hippocampal slices *ex vivo*, in an epileptic/pathological scenario as observed *in vivo*.

### Platinum Nanoparticles Do Not Negatively Impact Cell Viability in Normal or Pathological Conditions

Neural cell viability and neuronal cell integrity upon incubation with PtNPs were evaluated thorough PI uptake and β3-tubulin immunofluorescence, respectively, in normal and pathological conditions.

Before NPs incubation, a subset of slices was first challenged with KA for 24 h, as previously assessed to induce neurodegeneration, especially in the CA3 region. Application of PtNPs drop by drop on each slice was carried out to provide a homogeneous and direct deposition throughout the whole tissue surface to assess the different regional responses to a known NP concentration. After, slices were incubated with PtNPs for an additional 24-h period (Figure 1A). Image analysis and quantification of PI uptake was carried out in the whole slice (data not shown) and in the CA3, CA1, and DG regions. Our results show that PtNPs do not reduce cell viability in normal conditions (Figure 2A), as no significant differences were observed when compared to control cultures. In KA-treated slices, as already described above, KA at low concentrations (7 μM) for 24 h caused visible neuronal cell death in CA3 and, to a lower extent, in CA1 and DG regions compared to untreated cultures (Figure 2A). Despite this evident compromised tissue, no significant PI uptake was observed in KA-treated slices incubated with PtNPs in all the three regions (Figure 2B).

**FIGURE 3:**
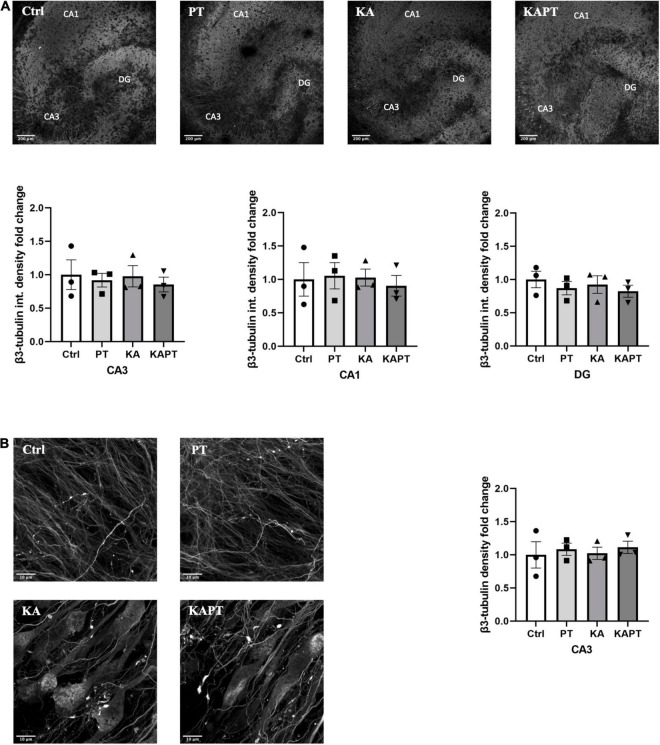
Platinum nanoparticles (PtNPs) do not further impact neuronal integrity in normal and pathological conditions after 24 h of contact. **(A)** Representative beta-3 tubulin immunostaining of organotypic hippocampal slices under control conditions (untreated), PtNPs, kainic acid (KA) treatment, and KA treatment + PtNPs. Bottom charts represent the quantitative analysis of beta-3 tubulin fluorescence intensity in CA3, CA1, and dentate gyrus (DG). **(B)** Magnified images of CA3 region showing alterations in microtubule morphology only in KA-treated slices compared to controls, regardless of PtNPs incubation. Right chart represents the quantification of the b3-tubulin immunostaining in the high magnification CA3 area for the different slices. Data are expressed as integrated density fold change from the basal value of control slices and presented as mean ± standard error of the mean (SEM) (*n* = 3 independent experiments; 4–6 slices analyzed per group/experiment). Tukey’s test after one–way analysis of variance (ANOVA) was used for statistical analysis.

To further characterize the impact of PtNPs on neuronal cells, we performed immunofluorescence analysis using β3-tubulin staining. Fluorescence intensity analysis of CA3, CA1, and DG areas was first performed to assess the effects of PtNPs on neuronal cells (Figure 3A). Our results show that PtNPs did not cause significant neuronal cell loss in normal conditions. Furthermore, we did not observe alterations in β3-tubulin fluorescence intensity in KA-treated slices in all the three regions (Figure 3A). In addition, we performed the analysis of β3-tubulin density at the level of the CA3 region. This region was selected as the area mainly susceptible to cellular alterations *in vivo and ex vivo* (Yu et al., 2016; Neville et al., 2018). Our results show that no significant alterations occurred in neuronal density, indicating that PtNPs do not impair neuronal integrity in normal conditions. Even though in KA-treated slices we observed evident impairment in neuronal integrity and the presence of aberrating pyramidal neurons at the level of the CA3 region due to the KA treatment, we do not report any significant difference in β3-tubulin density regardless of the PtNPs incubation (Figure 3B).

### Platinum Nanoparticles Cause Microglia Activation in Organotypic Hippocampal Slices

To investigate the impact of PtNPs on glial cells, we performed immunofluorescence analysis on microglia and astrocytes.

**FIGURE 4:**
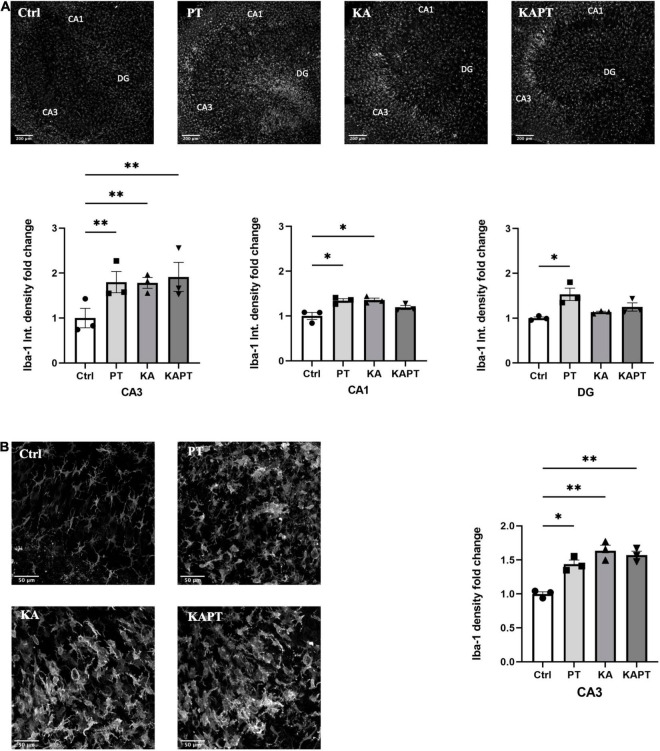
Platinum nanoparticles (PtNPs) cause microglia activation in organotypic hippocampal slices after 24 h of contact. **(A)** Representative Iba-1 immunostaining of organotypic hippocampal slices under control conditions, PtNPs, kainic acid (KA) treatment, and KA treatment + PtNPs. Bottom charts represent the quantitative analysis of Iba-1 fluorescence intensity in CA3, CA1, and dentate gyrus (DG). **(B)** Magnified images of Iba-1 stained microglia at the level of the CA3 region, showing altered morphology of cells in all the treatment groups compared to the control. Right chart represents Iba-1 density quantification at the CA3 region for all experimental groups. Data are expressed as Iba-1 fluorescence intensity and density fold change from the value of control slices and presented as means ± standard error of the mean (SEM) (*n* = 3 independent experiments; 4–6 slices analyzed per group/experiment). Tukey’s test after one–way analysis of variance (ANOVA) was used for statistical analysis. *p < 0.05; ^**^p < 0.01.

Iba-1 fluorescence intensity measurements were performed on CA3, CA1, and DG regions. Interestingly, we observed a significant increase in Iba-1 fluorescence intensity in slices incubated with PtNPs in all the three regions. In more detail, we report a significant 1.4-fold increase in Iba-1 fluorescence intensity in CA1 region, twofold increase in CA3 and 1.5-fold increase in DG area (Figure 4A). In KA-treated cultures, we reported higher Iba-1 intensity levels in CA3 (twofold increase) and, to a lower extent, in CA1 area (1.4-fold increase) (Figure 4A). Incubation of PtNPs in KA treated slices led to the same increase in Iba-1 as in the slices only subjected to KA treatment. Further analysis on microglia density was carried out in the CA3 area in all experimental groups. In normal conditions, we observed a significant twofold increase in microglial density after PtNPs incubation, as a result of cell activation toward a reactive phenotype (Figure 4B). As already reported in the literature, a higher increase in microglial density was observed in KA treated slices (Araki et al., 2020). However, consistent with the above-described data, incubation with PtNPs did not cause any exacerbation of the effect already induced by KA.

**FIGURE 5:**
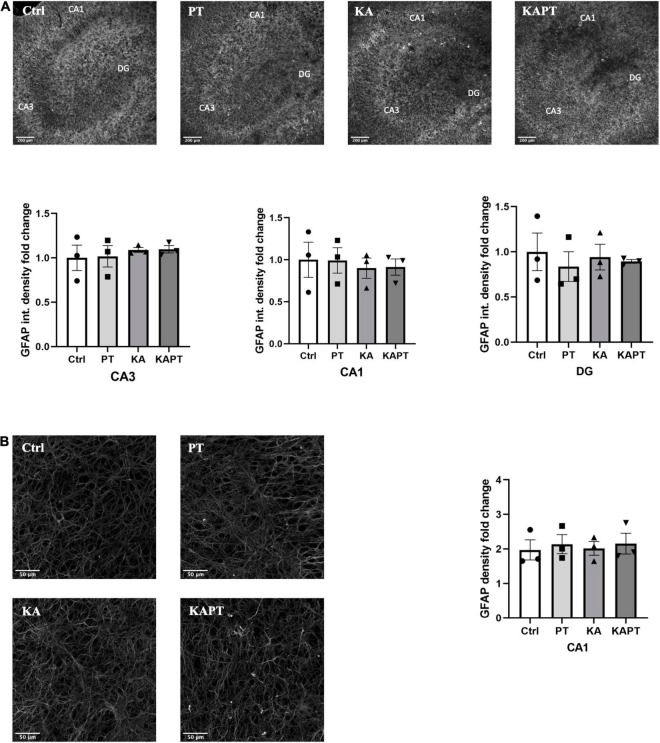
Platinum nanoparticles (PtNPs) do not cause astrogliosis in organotypic hippocampal slices after 24 h of contact. **(A)** Representative GFAP immunostaining of organotypic hippocampal slices under control condition, PtNPs, kainic acid (KA) treatment, and KA treatment with PtNPs. Bottom charts represent the quantitative analysis of GFAP fluorescence intensity in CA3, CA1, and dentate gyrus (DG). **(B)** Magnified images of GFAP stained astrocytes at the level of the CA1 region. Right chart represents GFAP density quantification at the CA1 region showing non-significant differences in all the treatment groups. Data are expressed as integrated density and density fold change from the value of control slices and presented as means ± standard error of the mean (SEM) (*n* = 3 independent experiments; 4–6 slices analyzed per group/experiment). Tukey’s test after one–way analysis of variance (ANOVA) was used for statistical analysis.

To investigate the reactivity of astrocytes to PtNPs, we performed GFAP immunofluorescence staining. GFAP fluorescence intensity measurements in CA3, CA1, and DG regions revealed non-significant alterations in astrocyte states in all the conditions tested (Figure 5A). Analysis of GFAP density was carried out at higher magnification in CA1 region as the most susceptible region to GFAP alteration in KA animal models of epilepsy (Vargas et al., 2013; Wu et al., 2021). In accordance with the measurements of GFAP fluorescence intensity, no significant differences were observed in astrocytes density (Figure 5B) in all the treatment groups. These data indicate that, despite having observed microglial activation after PtNPs administration in all the hippocampal regions, astrocytes density and integrity remained preserved after 24 h of contact with PtNPs.

### Platinum Nanoparticles Cause Morphological Alterations of Microglia

**FIGURE 6:**
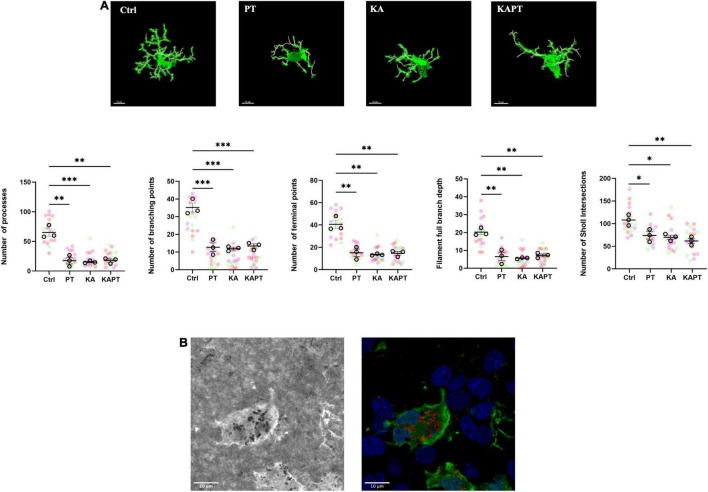
Platinum nanoparticles (PtNPs) cause morphological alterations of microglia after 24 h of contact. **(A)** Representative Iba-1 immunostaining of microglia at 63X magnification, showing alteration in microglia morphology in all the treatment groups compared to controls. White cylinders highlight branch complexity. Bottom charts represent microglia morphology quantification showing significant differences in the number of processes, branching and terminal points, as well as the branch complexity and ramification in all the treatment groups compared to controls. **(B)** Left: representative brightfield image showing PtNPs internalization by microglia. Right: merge of the brightfield image with PtNPs colored in red with the fluorescent image showing microglia in green and nuclei in blue. Dots in the dot plots represent individual cells. Data are presented as means ± standard error of the mean (SEM) (*n* = 3 independent experiments; 5–8 cells analyzed per group/experiment). Tukey’s test after one–way analysis of variance (ANOVA) was used for statistical analysis. *p < 0.05; ^**^p < 0.01; ^***^p < 0.001.

Further image analysis was carried out to study the impact of PtNPs on microglia morphology. Iba-1-stained microglia from CA3 region were imaged and analyzed using the filament tool on Imaris software. In both normal and pathological conditions, a significant reduction in the number of processes was observed in slices incubated with PtNPs (*p* < 0.01), accompanied by a reduction of the number of branching points (*p* < 0.001) and terminal points (*p* < 0.01) (Figure 6A). To analyze the degree of arborization of microglia, we also quantified the filament full branch depth (the highest value of bifurcations of the processes for each cell) and performed automated Sholl analysis in Imaris. We observe a significant decrease in the level of bifurcation in slices treated with PtNPs, indicating that microglia underwent morphological alterations from ramified state to a reactive ameboid state as result of cell activation. Moreover, we identified internalization of PtNPs by microglia and this could explain the reactive phenotype that was observed (Figure 6B). In KA-treated slices we reported a similar behavior of microglia in response to the induced neuronal cell death and neurodegeneration. It has been already reported *in vivo*, and *ex vivo* that microglia migrate at the level of the CA3 region in response to the neurodegeneration induced by KA acquiring a reactive ameboid shape (Araki et al., 2020). In line with the previous quantifications, we did not observe any amplification of the inflammatory response in KA-treated slices incubated with PtNPs.

## Discussion

Despite the improvements in nanobiomedicine and biomedical device fabrication, there are still significant limitations on biocompatibility and electrochemical performance of materials and coatings used for microelectrode design. Therefore, one of the current challenges in the field has been uncovering the most effective intervention strategies to overcome adverse biocompatibility and durability issues and to promote material homeostatic integration in the central nervous system. Toward this end, the use of appropriate experimental models can provide significant advantages in the development and testing of biocompatible smart materials for microelectrode design.

The main goal of this work was to test the biocompatibility and the acute cellular response to metal NPs, by exploring rodent brain organotypic cultures as *ex vivo* models. Among the great variety of *in vitro* biological models available, organotypic cultures represent one of the best biological tools for this purpose because they offer the possibility to mimic several pathological conditions and to isolate specific cerebral regions, ensuring the preservation of tissue architecture and synaptic organization (Gulino et al., 2019). Moreover, the tunability of the experimental conditions make them a relevant alternative for testing diverse new materials and designs at the preclinical level resulting in a refinement of subsequent *in vivo* studies and a reduction on the use of animal models.

We, in fact, showed through this work, the organotypic cultures versatility for the assessment of biocompatibility of materials such as Pt in physiological and induced pathological conditions. Considering the increasing number of biomedical applications of PtNPs, it is paramount to unravel their biocompatibility and toxicological potential while envisaging its safe clinical use. To the best of our knowledge, this is the first time that such organotypic based disease model has been explored to assess the biological performance of PtNPs. Such 3D complex models can serve as promising alternatives to animal studies used for investigation on the impact of metallic nanoparticles and devices on neural tissue. Additionally, glial cells, the effective determinants of the nervous tissue response to materials can be studied in detail in their 3D environment. Microglia are the key-regulators of brain inflammation and play a double edged-sword role in many pathophysiological processes. In the process of nervous tissue response, they act as first mediators of the acute phase of the response and become activated in the first hours after material contact. They reach the vicinity of the injury site and secrete pro-inflammatory cytokines which in turn lead to astrocyte and oligodendrocyte activation along with progressive neuronal cell loss at the level of the tissue-material interface (Gulino et al., 2019; Wellman et al., 2019; Huang et al., 2020).

In this study, we report that short time (24 h) incubation of organotypic cultures with monodisperse citrate-coated PtNPs did not hamper neuronal viability and integrity. PI uptake analysis revealed absence of cell viability impairment, as well as similar β3-tubulin fluorescence expression, density and morphology indicating the absence of neuronal cell loss. However, we reported a significant increase in Iba-1 fluorescence expression and density in CA3, CA1, and DG areas as consequence of microglial cell activation (Kozai et al., 2012; Yang et al., 2021). Morphological analysis revealed that microglia displayed a unramified morphology, with reduced number of branches and process length, indicating the acquisition of a reactive ameboid phenotype.

The impact of PtNPs was also investigated in organotypic cultures under excitotoxic conditions induced by KA. It is widely described in the literature that KA *in vivo* and *ex vivo* induces neuronal cell loss, hippocampal neurodegeneration, glial activation and oxidative stress. To mimic the same pathological scenario, typical of neurological conditions like for example epilepsy, we confirmed through microscopic analysis the induction of neural cell cytotoxicity, neuronal degeneration and microglia activation mainly at the level of the CA3 region. Surprisingly, we did not report any exacerbation of the response of KA-treated slices to PtNPs incubation for 24 h. Such finding may be explained by the considerable cytotoxic effect induced by KA that could not be significantly added up by the PtNPs action, as observed in non-pathological conditions. Another hypothesis that can explain the non-observation of an additive effect could result from neuroprotection elicited by microglia in the pathological tissue explant. So, when the addition of the PtNPs occurs, the inflammatory response triggered by the KA treatment is already ongoing and, therefore, the tissue reaction to the PtNPs is distinct from the one observed in the physiological tissue explant. In fact, it has been previously described that microglia can play a neuroprotective role in hippocampal excitotoxicity (Vinet et al., 2012). In particular, it has been reported an increase in neuronal survival rate *in vitro* (Yu et al., 2019; Wang et al., 2021), as well as upregulation of pro- and anti-inflammatory genes after intranasal administration of KA *in vivo* (Sabilallah et al., 2016). In light of these observations, our findings may support the hypothesis of a non-detectable effect and/or a neuroprotective effect exerted by microglia after 24 h of incubation of KA and could explain the absence of an additive effect in the response to PtNPs as observed in normal conditions. Finally, our experiments demonstrated the importance of screening materials in biological models with increased complexity that closely mimic the *in vivo* scenario and strongly point to the importance of the choice of relevant based disease models for a proper material characterization in homeostatic and non-homeostatic conditions.

In this work we employed rodent brain organotypic cultures as *ex vivo* models to study the biocompatibility of new classes of smart materials and coatings for microelectrode design to be applied in disease context. We in fact showed, through our work, their ability for the assessment of biocompatibility of PtNPs and the possibility to evaluate the glial and neuronal acute cell response under reproducible and controlled experimental conditions. Nevertheless, due to the possibility to maintain organotypic cultures for extended periods of time, one can also envisage the investigation of the implications of PtNPs in the brain tissue in the long term, what will be the focus of future studies in the group.

## Data Availability Statement

The raw data supporting the conclusions of this article will be made available by the authors, without undue reservation.

## Ethics Statement

The animal study was reviewed and approved by the Animal Welfare and Ethics Review Body of the Instituto de Investigação e Inovação em Saúde (i3S).

## Author Contributions

MG was responsible for material preparation and data collection, and wrote the first draft of the manuscript. AP, SS, and MG contributed to the scientific analysis of the results. AP and SS performed the review and editing. AP was responsible for acquisition of the financial support for the project leading to this publication. All authors contributed to the conceptualization and design of the study and read, and approved the final submitted version.

## Conflict of Interest

The authors declare that the research was conducted in the absence of any commercial or financial relationships that could be construed as a potential conflict of interest.

## Publisher’s Note

All claims expressed in this article are solely those of the authors and do not necessarily represent those of their affiliated organizations, or those of the publisher, the editors and the reviewers. Any product that may be evaluated in this article, or claim that may be made by its manufacturer, is not guaranteed or endorsed by the publisher.
